# Health-Related Internet Use in People With Cancer: Results From a Cross-Sectional Study in Two Outpatient Clinics in Sweden

**DOI:** 10.2196/jmir.6830

**Published:** 2017-05-15

**Authors:** Susanne Mattsson, Erik Martin Gustaf Olsson, Birgitta Johansson, Maria Carlsson

**Affiliations:** ^1^ Department of Public Health and Caring Sciences Uppsala University Uppsala Sweden; ^2^ Department of Women’s and Children’s Health Uppsala University Uppsala Sweden; ^3^ Department of Immunology, Genetics and Pathology, Experimental and Clinical Oncology Uppsala University Uppsala Sweden

**Keywords:** Oncology, eHealth, support

## Abstract

**Background:**

The access to various forms of support during the disease trajectory is crucial for people with cancer. The provision and use of Internet health services is increasing, and it is important to further investigate the preferences and demographical characteristics of its users. Investigating the uptake and perceived value of Internet health services is a prerequisite to be able to meet the needs in the targeted group.

**Objective:**

The objective of this study was to investigate health-related Internet use among people with cancer.

**Methods:**

The health online support questionnaire (HOSQ), examining the incentives for health-related Internet support use, was administered in two Swedish outpatient hospital clinics. Of the 350 copies of the questionnaire handed out, 285 (81.4%) were returned, answered by persons with cancer who had completed treatment or were under active surveillance or another medical treatment.

**Results:**

A total of 215 (76.2%, 215/282) participants reported Internet use since being diagnosed with cancer. Internet-users were younger (*P*<.001), more likely to have a partner (*P*=.03), and had a higher level of education than nonusers (*P*<.001). The most common health-related activity on the Internet was searching for information (77.2%, 166/215), and users searched significantly more immediately after diagnosis compared with later on (*P*<.001). Use of My Healthcare Contacts was considered the most valuable Internet activity. Having a university degree (*P* ˂.001) and being younger in age (*P*=.01) were associated with a significantly higher frequency of health- related Internet use.

**Conclusions:**

People with cancer turn to the Internet for informational support that enables them to influence their care and to stay in touch with friends and relatives. Demographical differences regarding the uptake of Web-based support remains. This indicates a need for research on how to bridge this digital gap. By learning more about the use of health-related support on the Web among people with cancer, adequate support can be offered and potential strain reduced.

## Introduction

The treatment of cancer has gone through some important changes the past decades and one of them is an increase in outpatient care services coupled with a decrease in inpatient care [1,2]. This implies that patients spend less time in hospital, which may result in a decrease in various kinds of support delivered by health care staff and peer patients. Increased outpatient care may be beneficial for patients who can spend less time at the hospital and even from an economic perspective, but presents challenges regarding the coordination of cancer care [3]. At the same time, the development toward a more empowered, self-determined, and partaking patient is continuing [4].

Internet-based technologies such as patient portals, websites, and apps managed by health care institutions, have been recognized as a significant lever to improve cancer care coordination [5]. Internet delivered support may also be a tool to increase patient empowerment [6] and has been found as cost effective as well as an important factor in reducing the need for support from the health care system [7].

The use of the Internet as a source of support is a trend that has increased rapidly among cancer patients during the past decade [8]. Motives for using the Internet as a source of support among patients with cancer are ease of communication and access to the most up-to-date information and peer support [9,10]. The Internet offers a wide range of websites delivering different kinds of support. In addition to searching for health information on the Web, people with cancer visit online peer support networks, blogs, and social networks [11,12]. This has been found to be a valuable source of social support [13]. Social support is associated with a better health-related quality of life, fewer stress symptoms, and better health [14-16], and might reduce anxiety and depression symptoms and increase quality of life in people with severe diseases such as cancer [17,18].

The access to the Internet in Europe has increased significantly during the past decades and is high in the Northern countries [19]. However, health-related Internet use is affected by sociodemographic characteristics such as age, gender, education, and civil and socioeconomic status, which should be further addressed in future research [20]. More knowledge is needed regarding cancer patients’ current use and appreciation of the Internet as a tool for health-related support, since these patients are generally older which indicate a barrier for Internet use [3,20].

Due to potential long lasting effects of the disease and treatments, people with cancer are at a higher risk for comorbidities and psychosocial problems throughout their life [21]. An understanding of how this heterogeneous group uses the Internet for available health-related support may increase the possibilities to offer adequate interventions of Internet-based support to alleviate potential distress.

This study aims to investigate health-related Internet use and the perceived value of this among people with cancer. A further aim is to describe the incentives for Internet use and to explore associations between Internet use and medical and demographic variables.

## Methods

### Sample and Procedure

A convenience sample of persons with cancer was included from November 2014 to February 2015. Subjects were consecutively recruited at a scheduled visit to an oncology or urology outpatient clinic at a university hospital in Sweden. The inclusion criteria were an age of 18 years or older and completion of the initial treatment (surgery, chemotherapy, radiotherapy) or currently undergoing either hormone treatment, active surveillance or other medical treatment. This was in order for them to have gained some perspective on how they had used the Internet after being diagnosed. Exclusion criteria were inability to understand Swedish, cognitive impairment, or participation in an ongoing Internet-based intervention (U-CARE) at the hospital that could influence the reported use of support on the Internet [22]. Eligible patients were identified through the clinic visit list, approached, and handed the questionnaire in the waiting room. They were given oral and written information about the study and could choose to complete the questionnaire at the clinic or at home and return it by mail in a prepaid envelope. Consent to participate was implied by completion and return of the questionnaire.

Ethics approval was granted by the regional ethical review board in Uppsala (2013-11-20; diary number 2013/436).

### Data Collection

The first question in the questionnaire asked about whether the patients had used the Internet. The group of Internet users was defined as the group who reported that they had used the Internet on a computer, mobile phone or a tablet, at least once or twice since being diagnosed with cancer. Internet users were asked if they had visited information sites; social media sites such as Twitter, Facebook, and Instagram; discussion forums and blogs regarding health, diseases, treatments, lifestyle or similar; and whether they had created their own blogs and/or discussion threads and/or commented on others. The questions were rated from 0=never, to 1=once or twice ever, 2=at least once a year, 3=at least once a month, 4=at least once a week, and 5=daily or almost daily. Subjects were further asked a question about the use of an eHealth service (My Healthcare Contacts) that allows patients to request, cancel, or reschedule appointments; read their medical record; and renew their prescriptions. This question was rated from 0=never, to 1=sometimes, and 2=several times. The questions regarding the frequency of using the apps were asked in relation to both the time immediately after diagnosis, and later on. All patients gave their own definition of how long the time immediately after diagnosis was. How valuable they considered the use of different apps to handle their health situation was, was rated on a scale ranging from 0=not valuable at all, to 10=very valuable.

The incentives for Internet use were investigated using the health online support questionnaire, (HOSQ) [23]. The HOSQ was developed and primarily tested in two Swedish samples (one nonclinical sample and one clinical cancer sample) [23]. The HOSQ is validated in Swedish and consists of 18 questions regarding the incentives for health-related Internet use. They are scored on a 6-point Likert scale describing the frequency of use ranging from 0=not relevant or never, to 5=on a daily basis, and the highest possible total score is 90 (Cronbach alpha=.92). The HOSQ can be divided into two subscales: the reading scale and the interacting scale. The subscales contain 9 questions each and the total possible score is 45 for each subscale (Cronbach alpha: reading=.88; interacting=.77). The initial validation study of the HOSQ depicted a response pattern revealing expected differences both between the interaction and reading scales and according to age, gender, education, and health problems, hence showed a good face- and construct validity [23].

Demographic data on age, sex, civil status (having a partner or being single), birth country, educational level, diagnosis, time since diagnosis, and cancer treatment (completed, hormone, active surveillance, other) were collected using project-specific questions answered by both Internet users and nonusers.

### Statistical Analysis

The statistical analyses were performed using IBM SPSS Statistics (version 20.0).

The level of significance in this study was *P* ≤.05.

Internet use, the perceived value of this, and the incentives for Internet use were analyzed descriptively. Statistical comparisons between groups were conducted with chi-square tests (sex, education, civil status, treatment, and birth country) and *t*-tests (age). Comparisons between the use of apps (information sites, discussion forums, blogs, and so on) immediately after diagnosis and later on were conducted with Wilcoxon signed rank test due to positively skewed data. Correlations between age and the HOSQ total score and the subscales were conducted with Spearman rho. The associations between Internet use and demographic and medical data were analyzed with multiple logistic regressions including age, sex, education, civil status, and completion of treatment as independent variables. This analysis was done on the HOSQ total scale and separately for each subscale. Due to positively skewed data, all HOSQ scales were dichotomized based on the median of the respective scale.

## Results

### Internet Use and Demographic Characteristics

In total, 350 questionnaires were handed out and 285 (81.4%, 285/350) were answered and returned. Three were excluded due to missing data. Two hundred and fifteen (76.2%, 215/282) of the participants reported Internet use after being diagnosed with cancer. Internet users were younger, more often had a partner, and had higher education compared with nonusers. The most common type of diagnosis was prostate or breast cancer and the median time since diagnosis was 3 years (Table 1).

### The Health Online Support Questionnaire (HOSQ) Scores Among the Internet Users

The median score of the HOSQ reading subscale was higher compared with the HOSQ interacting subscale median score (Table 2).

### Associations Between Web-Based Support and Demographic and Medical Variables

The multiple regression analysis showed that lower age was significantly associated with higher score on HOSQ total (*P*=.01) and the reading subscale (*P* ˂.001). Having a university degree was significantly associated with a higher score on all three scales (reading: *P*=.01; interacting, *P=*.05; Total: *P* ˂.001) compared with elementary school. Completion of treatment was significantly associated with a lower score on the HOSQ reading subscale (*P=*.05; Table 3).

### Incentives for Internet Use

The primary incentives for using the Internet for health-related support was to search for information that could improve the overall health, enable more informed decisions, and to get the best possible health care. Instrumental support such as searching for scheduled appointments, addresses, or phone numbers to health care was also reported by many Internet users. The most reported incentives from the interactive scale was staying in touch with friends and colleagues and reading about other peoples’ experiences of a similar situation (see Table 4).

**Table 1:** Demographic characteristics of Internet users (n=215) and nonusers (n=67).

Demographic and medical characteristics		Internet users, n=215	Nonusers, n=67		*P* value
Age (years): mean (SD), range		60.5 (13.8) 20-84	70 (8.9) 39-90		<.001
**Sex^a^, n (%)**				.44	
	Male	119 (55.3)	35 (52.2)		
	Female	94 (43.7)	32 (47.7)		
**Education^a^, n (%)**				<.001	
	Elementary school^b^	34 (15.8)	31 (46.3)		
	Secondary school	72 (33.5)	21 (31.3)		
	University	105 (48.8)	13 (19.4)		
**Civil status^a^, n (%)**				.03	
	Having a partner	181 (84.2)	48 (71.6)		
	Single	31 (14.4)	17 (25.4)		
Birth country: Sweden		189 (87.9)	57 (85.0)	.94	
**Diagnosis^a^, n (%)**					
	Prostate cancer	69 (32.0)	16 (23.8)		
	Breast cancer	34 (15.8)	12 (17.9)		
	Gastro-intestinal cancer	15 (6.9)	7 (10.4)		
	Malignant melanoma	11 (5.1)	4 (5.9)		
	Lung cancer	2 (0.9)	7 (10.4)		
	Lymphoma	30 (13.9)	6 (8.9)		
	CNS^c^tumor	12 (5.6)	1 (1.5)		
	Gynecological cancer	8 (3.7)	1 (1.5)		
	Head and neck cancer	2 (0.9)	2 (2.9)		
	Sarkoma	9 (4.2)	1 (1.5)		
	Hematological cancer	4 (1.9)	0 (0.0)		
	Other cancer	6 (2.8)	2 (2.9)		
Time since diagnosis, median, (range), years		3 (1-50)	3 (1-29)		
**Treatment condition^a^, n (%)**				.17	
	Completed treatment	127 (59.1)	35 (52.2)		
	Hormone treatment	37 (17.2)	16 (23.9)		
	Active surveillance	18 (8.4)	2 (2.9)		
	Other treatment	26 (12.1)	12 (17.9)		

^a^Due to occasional missing data in the questionnaires, the sum of the subgroups may be lower than the corresponding total numbers of individuals.

^b^1-9th grade.

^c^CNS: central nervous system.

**Table 2:** The median and interquartile range of health online support questionnaire (HOSQ) scores among the Internet users (n=215).

Demographic and medical characteristics		Total (Max: 90)		HOSQ^a^ reading (Max: 45)		HOSQ interacting (Max: 45)	
		Median	Interquartile range	Median	Interquartile range	Median	Interquartile range
All		12	19	8	15	2	6.75
**Sex**							
	Male	9	16.25	7	13	1	4
	Female	14.5	20	8	14.5	4	10
**Education**							
	Elementary school	7	15	4	8.75	1	4
	Secondary school	7	16	6	10	1.50	5
	University	16	20	11	14	3	10
**Civil status**							
	Having a partner	12	28.75	8	13.75	2	6
	Single	12	24.5	8	16.5	1	8
**Completed treatment**							
	Yes	12	22	7	16	2	7
	No	12	17.5	9	14	2.5	7

^a^HOSQ: health online support questionnaire.

**Table 3:** Multiple regression analyses of the health online support questionnaire (HOSQ) scores and demographic and medical variables in the group of Internet users (n=215).

Demographic and medical variables		Total		HOSQ^a^ reading		HOSQ interacting	
		OR^b^	CI	OR	CI	OR	CI
Age, years		0.96	0.94-0.99	.95	0.93-0.98	0.99	0.96-1.01
**Sex**							
	Female (ref)						
	Male	0.59	0.41-1.67	1.14	0.57-2.30	0.69	0.35-1.36
**Education**							
	Elementary school (ref)						
	Secondary school (1)	1.58	0.55-4.54	1.71	0.59-4.91	1.58	0.61-4.11
	University (2)	5.37	1.98-14.55	4.74	1.74-12.9	2.75	1.12-6.77
**Civil status**							
	Having a partner (ref)						
	Single	0.99	0.40-2.43	1.07	0.43-2.66	0.59	0.25-1.38
**Completed treatment**							
	Yes (ref)						
	No	1.74	0.85-3.54	2.03	1.01-4.11	1.11	0.57-2.15

^a^HOSQ: health online support questionnaire^.^

^b^OR: odds ratio.

**Table 4:** The frequency of participants reporting never, once or twice ever, or more than sometimes on the items respectively of the health online support questionnaire.

Item		Never n (%)	Once or twice ever n (%)	More than sometimes n (%)
**“Since I was diagnosed with cancer I have used the Internet...”**				
	To search for information that can improve my overall health (R^a^)	60 (27.9)	63 (29.3)	81 (37.7)
	To search for scheduled appointments, addresses or phone numbers to health care providers (R)	76 (35.3	59 (27.4)	65 (30.2)
	To be able to make more informed decisions regarding my illness or health condition (R)	78 (35.3)	59 (27.4)	64 (29.8)
	To search for the very latest research regarding my health situation (R)	84 (39.1)	51 (23.7)	65 (30.2)
	To search for information so I can better understand physicians and other health care personnel (R)	92 (42.8)	57 (26.5)	52 (24.2)
	To search for information from various sources so I can get the best possible health care (R)	98 (45.6)	44 (20.5)	66 (30.7)
	To seek further information when I feel worried (R)	98 (45.6)	41 (19.1)	61 (28.4)
	To read about other people’s experience of a particular illness or health condition or treatment (R)	106 (49.3)	44 (20.5)	50 (23.3)
	To find out whether symptoms I have discovered are dangerous or not (R)	107 (49.8)	46 (21.4)	47 (21.9)
	To keep friends and relatives informed about how I’m feeling (I^b^)	111 (51.6)	36 (16.7)	53 (24.7)
	To stay in touch with friends and colleagues when I’m sick or not feeling well (I)	114 (53.1)	25 (11.6)	59 (27.4)
	To get feedback from friends and relatives on how I’m handling my illness or health situation (I)	143 (66.5)	28 (13.0)	27 (12.6)
	To get feedback from people who have or have had the same health problem as I have (I)	150 (69.8)	28 (13.0)	20 (9.3)
	To talk about a treatment for an illness or health condition that I’ve been through (I)	152 (70.7)	20 (9.3)	27 (26.5)
	To share practical advice and suggestions about illness or health (I)	154 (71.6)	23 (10.7)	22 (10.2)
	To express my opinion regarding health or illness or care (I)	165 (76.7)	17 (9.9)	16 (7.4)
	To look for compassion when I’m not feeling well (I)	170 (79.1)	13 (6.1)	16 (7.4)

^a^R: reading scale.

^b^I: interacting scale.

### The Use of Apps on the Internet

Among the Internet users, 166 (77.2%, 166/215) reported that they had used the Internet to search for health information. Forty five (20.9%, 45/215) had visited health-related discussion forums, 39 (18.1%, 39/215) had visited blogs, only one (0.5%, 1/215) had taken part in psychological treatment on the Internet, 73 (33.9%, 73/215) reported use of social media. My Healthcare Contacts had been used by 82 (38.1%, 82/215) and 38 (17.7%, 38/215) of them had taken part of information and test results in the medical e-record services, 8 (3.7%, 8/215) had scheduled appointments, 12 (5.6%, 12/215) had renewed prescriptions, and 5 (2.3%, 5/215) had chosen a general practitioner (GP). My Healthcare Contacts was considered the most valuable Internet health resource (mean=6.7; standard deviation [SD]=3) compared with information sites (mean=6.2; SD=2.6), forums (mean=6.1; SD=2.9), blogs (mean=5.8; SD=3.2), and social media (mean=4.1; SD=3.3).

The daily use of information apps immediately after diagnosis was higher (21.9%, 47/215) than later on (5.1%, 11/215), *P*<.001. One hundred and twelve (52.1%, 112/215) considered the length of the time immediately after diagnosis to be somewhere between 1 day and 3 months. The total range of reports was 1 day to 5 years and the median time was 3 months. No differences were found regarding use of other apps than information between the time immediately after the diagnosis and thereafter. The vast majority of users visiting information sites (86%) and discussion forums (81%) did so regarding their own health. Use of the other apps was less related to their own health (blogs 58%; social media 28%).

## Discussion

### Principal Findings

This study found that the persons with cancer who use the Internet for health-related support mainly search for information that enables them to improve their overall health and get the best possible health care. The health care delivered tool My Healthcare Contacts was considered very valuable among the ones who used it, hence, seems to be a satisfactory app on the Web. Younger and higher educated used the Internet significantly more than the older and less educated.

Information sites were the most frequented sites compared with social media, discussion forums, and blogs. This finding converges with other studies on health-related Internet use [9,20,24]. Reported reasons in previous studies are perceived lack of information from health care, the efficiency of the Internet, and as also found in this study, a desire to stay updated on the most recent information about disease-related matters [5,8,24]. It is well known that the need for information is associated with health-related variables [11]. This study found that the search for information was significantly higher during the time immediately after diagnosis than later. Hence, it is of major importance for the health care system to provide patients with adequate informational support during this phase. As this study shows, information found on the Internet is considered valuable. It has been reported that well-informed patients report greater engagement in care decisions and an increased confidence in their interactions with health care providers [24].

Peer support is often referred to as an important factor for people with cancer, even though it is not considered as one of the most important health activities on the Internet [3]. The use of blogs and discussion forums was not that high in this study even though the development of these apps as well as their use has increased over the past decade [25]. The relatively low frequency, in this study, of visiting these sites could be explained by the relatively high age in this group since older patients are less likely to use Web-based tools [3]. The score on the HOSQ interacting scale was lower than the score on the reading scale, which corroborates other studies reporting that taking part of information rather than also sharing information is more common [26,27]. It could also be that this group prefers face-to-face support since only a third of the Internet users reported use of social media. By addressing cancer patients’ preferences and providing customized support in navigating the Internet, which has been found necessary, the Internet might become a significant source of peer support for this group.

The demographic factors found to predict Internet use in this study were young in age and having a university education. The variables that frequently appear to influence health-related Internet use are age, gender, educational level, perceived health, and socioeconomic and civil status [3,20,28]. According to the unified theory of acceptance and use of technology (UTAUT), there are factors such as gender and age that mediate actual usage of technology [29]. In this study, still being under treatment or active surveillance predicted a higher frequency of reading on the Internet. Other research has shown that there is also a need for support after a patient have finished treatment and are in clinical remission, at which point the contact with the health care decreases [30]

One third of the Internet users reported use of My Healthcare Contacts, where taking part of information and finding out test results in the medical e-record services, was the most frequent activity. This was the app that was reported as the most valuable. There are findings suggesting that the majority of cancer patients consider improved access to their medical records as something that should be prioritized [3] Scheduling appointments, renewing prescriptions, and choosing one’s GP was also reported, which can be highly efficient for patients as well as the health care system in reducing the workload.

Despite just over 20 years of Internet, the number of people using it is still expanding in Sweden. The access as well as Internet use is still increasing even though the vast majority of people in Sweden already use the Internet. In particular, the group of younger pensioners using the Internet is growing. Currently, almost 100% of people between the ages of 12-55 years, and approximately 90% of people aged 60-65 years use the Internet in Sweden [31]. Future patients with cancer will be more active Internet users compared with today’s patients. This puts demands on the health care system regarding the development of relevant Web apps to meet their needs.

### Strengths and Limitations

The response rate was high (81%) and 75% of the participants were Internet users. To avoid selection bias the questionnaire was handed out at the clinic instead of being administered in a Web-based context to Internet users only. This way we were able to compare demographic and disease-related variables between the Internet users and the nonusers. The heterogeneity regarding diagnosis, age, educational level, and sex was satisfactory. Thus, the results may be fairly representative for people with cancer but limited to patients that have completed their initial treatment. However, there is a need for diagnose specific studies as well.

Many of the patients who chose not to answer the questionnaire said that they did not use the Internet, hence, did not think it was relevant for them to respond. Therefore, the percentage of Internet users in this study may be higher than what is representative for the population examined.

The patients’ definition of the time immediately after the diagnosis varied greatly. Therefore, results showing that the need for information was significantly higher immediately after diagnosis compared with later should be interpreted with caution, since the time “immediately after” the diagnosis might overlap the time “later on.”

A recall bias among the Internet users should also be taken into consideration since the median time since diagnosis is 3 years, meaning that the frequencies of Internet use may be under- or overestimated, which decreases the reliability of the findings.

The questionnaire used in this study, the HOSQ, has not been used previously, so the validity and reliability are uncertain. However, it has been psychometrically tested and validated regarding face and content validity in two samples where the response pattern revealed expected differences both between the interaction and reading scales and according to age, gender, education, and health problems [23].

According to UTAUT [29], there are factors mediating actual usage of technology that has not been collected in this study. It should therefore be taken into consideration that there may be other variables than the one collected that may have an impact on the results in this study.

### Conclusions

This study has found that patients turn to the Internet primarily for informational support that enable them to improve their overall health, make more informed decisions, and to get the best possible health care. Also to stay in touch with friends and colleagues and take part of other peoples’ experiences of a similar situation. Instrumental support such as searching for scheduled appointments, phone numbers, and addresses to health care was also something that they used the Internet for. The perceived value of the apps examined in this study was generally high. The use of instrumental support such as My Healthcare Contacts was considered the most valuable activity on the Web. Internet use was associated with having a university degree and being younger. This may indicate that the threshold for health-related activities on the Web is higher for older and less educated individuals, which needs to be addressed in future research.

### Practice Implications

A better understanding of health-related Internet use in different groups is a prerequisite to the provision of adequate Internet delivered support. By learning more about the incentives for health-related Internet use in various contexts of people with cancer, we may be able to develop tailored support that may alleviate potential distress, save costs, and reduce health care workload.

## Abbreviations

HOSQ: health online support questionnaire
